# Residue and Dietary Risk Assessment of Chiral Cyflumetofen in Apple

**DOI:** 10.3390/molecules23051060

**Published:** 2018-05-02

**Authors:** Jing Guo, Minmin Li, Yongguo Liu, Fengzhong Wang, Zhiqiang Kong, Yufeng Sun, Jia Lu, Nuo Jin, Yatao Huang, Jiameng Liu, Frédéric Francis, Bei Fan

**Affiliations:** 1 Institute of Food Science and Technology, Chinese Academy of Agricultural Sciences/Key Laboratory of Agro-products Quality and Safety Control in Storage and Transport Process/Laboratory of Agro-products Quality Safety Risk Assessment, Ministry of Agriculture, Beijing 100193, China; 13520410391@163.com (J.G.); liminmin@caas.cn (M.L.); wangfengzhong@sina.com (F.W.); sunyufeng@caas.cn (Y.S.); luciatime@foxmail.com (J.L.); jinnuo@caas.cn (N.J.); huangyatao@caas.cn (Y.H.); ljiam88@sina.com (J.L.); 2 Functional and Evolutionary Entomology, Gembloux Agro-Bio-Tech, University of Liège, Passage des Déportés 2, 5030 Gembloux, Belgium; frederic.francis@ulg.ac.be; 3 Beijing Key Laboratory of Flavor Chemistry, Beijing Technology and Business University (BTBU), Beijing 100048, China; liuyg@th.btbu.edu.cn

**Keywords:** chiral cyflumetofen, enantioselective degradation, distribution, ultra-performance convergence chromatography, dietary risk assessment

## Abstract

Ultra-performance convergence chromatography is an environmentally-friendly analytical method that uses dramatically reduced amounts of organic solvents. In addition, a robust and highly sensitive chiral separation method was developed for the novel chiral acaricide cyflumetofen by using ultra-performance convergence chromatography coupled with tandem mass spectrometry, which shows that stereoisomer recoveries determined for various apple parts ranged from 78.3% to 119.9%, with the relative standard deviations being lower than 14.0%. The half-lives of (−)-cyflumetofen and (+)-cyflumetofen obtained under 5-fold applied dosage equal to 22.13 and 22.23 days, respectively. For 1.5-fold applied dosage, the respective values were determined as 22.42 and 23.64 days, i.e., the degradation of (−)-cyflumetofen was insignificantly favored over that of its enantiomer. Importantly, cyflumetofen was unevenly distributed in apples, with its relative contents in apple peel, peduncle, and pomace equal to 50%, 22%, and 16%, respectively. The proposed method can be used to efficiently separate and quantify chiral pesticide with advantages of a shorter analysis time, greater sensitivity, and better environmental compatibility. Additionally, the consumption of apples with residue of cyflumetofen did not pose a health risk to the population if the cyflumetofen applied under satisfactory agricultural practices after the long-term dietary risk assessment.

## 1. Introduction

Apples are one of the major fruits in the world, with China being the largest producer [1,2]. Importantly, the apple peel exhibits a potent antioxidant activity and can greatly inhibit the growth of liver and colon cancer cells [3,4], which confirms the popular saying that an apple a day keeps the doctor away. However, apples and apple trees are vulnerable to pests and diseases, with a noteworthy example being *Tetranychus urticae*, or red spider, which inflicts serious damage to apple quality. To mitigate this problem, a large number of acaricides are used for insect and disease control, which is exemplified by cyflumetofen [2-methoxyethyl-(*RS*)-2-(4-*tert*-butylphenyl)-2-cyano-3-oxo-3-(α,α,α-trifluoro-*o*-tolyl)propionate]. This is a novel acaricide developed by Otsuka AgriTechno Co., Ltd. (Osaka, Japan) that is widely used in orchards for mite control [5]. However, the heavy use of pesticides in orchards has led to many problems, with pesticide contamination becoming one of the most important food safety issues.

**Figure 1:**
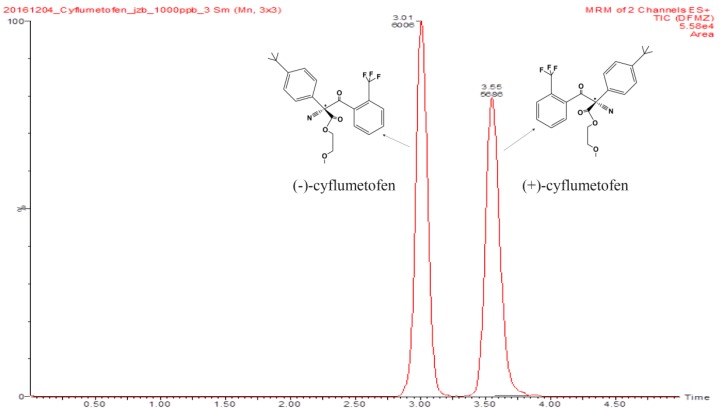
UPCC-MS/MS chromatograms of cyflumetofen enantiomers.

Commercial cyflumetofen is a racemic mixture of two enantiomers (see Figure 1) that was first registered in Japan in 2007 and is currently approved in 15 countries including China as an agent controlling the growth and reproduction of red spiders by inhibiting the mitochondria complex II [5,6]. The two enantiomers of cyflumetofen exhibit significant differences in toxicity, environmental fate, and bioactivity due to their different interactions with enzymes and other chiral biomolecules [7,8]. The toxicities of cyflumetofen to target mites and human cells are comparable, which deceases in the order of (−)-cyflumetofen > *rac*-cyflumetofen > (+)-cyflumetofen [9]. This means that the corresponding residues on apples pose a threat to human health [10]. In view of the above and the growing attention attracted by food security issues [11,12], including food contamination with pesticides [13], the investigation of cyflumetofen behavior in apples is a matter of high scientific significance.

Up to now, research on cyflumetofen enantiomers was mainly focused on developing methods for their separation and individual toxicity determination, such as those based on high-performance liquid chromatography [9] and ultra-performance convergence chromatography coupled with triple quadrupole mass spectrometry [14]. Since the concept of “green chemistry” has become more widely accepted [15,16], ultra-performance convergence chromatography coupled with triple quadrupole mass spectrometry (UPC^2^-MS/MS) has become an alternative and complementary method to the HPLC-MS/MS technique [17]. UPC^2^ integrates supercritical fluid chromatography (SFC) and ultra-high performance liquid chromatography (UHPLC) technologies [18]. As such SFC is a chromatographic technique in which a supercritical fluid such as supercritical CO_2_ is used as the mobile phase, with the advantages of low viscosity, high separation efficiency, environmental benefits [17]. UPC^2^ offers attractive features, including reduced run times, lower solvent consumption, and dramatically increased peak capacity and sensitivity [18]. Recently, several studies on the application of UPC^2^-MS/MS in separating and quantifying basic compounds [19], isomers [20], and bioactive compounds [21] have been reported. 

The mechanism of enantioselective cyflumetofen toxicity has already been characterized [6,22,23,24,25], e.g., cyflumetofen was shown to induce apoptosis, damage, and G1-phase cycle arrest in SH-SY5Y cells [26]. Hu et al. studied the dynamics of cyflumetofen residues in strawberries and performed the corresponding safety evaluation, which reveal that the highest residue content was observed for cyflumetofen suspension concentrate treatment (0.5882 mg/kg, half-life = 9.364 day) [27]. Liu, et al. studied the distribution of racemic cyflumetofen in home-canned tomatoes, which shows that the processing factor (PF) of tomato samples after each unit process was generally less than unity, whole especially that after the peeling process. This equalled 0.28 [28]. However, the environmental behavior of individual cyflumetofen enantiomers is only scarcely described [9], with the prevalence of studies dealing with racemic cyflumetofen, which poses a risk of inaccurately evaluating the threat posed by cyflumetofen residues to the environment and human beings [28]. Therefore, more research should be conducted to investigate the degradation of cyflumetofen enantiomers for a comprehensive risk assessment.

Cyflumetofen was used as a novel chiral acaricide on the apple. However, some surveys were conducted to determine the occurrence of cyflumetofen in different food products [29,30], especially in citrus, Yet, a few studies attempted to assess its potential risks to human health [31]. In addition, dietary risk assessment associated with cyflumetofen contamination in apple has never been reported previously in China, to our best knowledge. For food quality and safety, the higher residue levels of pesticides in food could not indicate the higher potential health risk of apples intake, because dietary exposure risks relate not only to the pesticide residue levels but also to the pesticide toxicity and food consumption data [32]. Therefore, determination of cyflumetofen in apple is urgently needed for assessing its food safety risks. 

Most of the previous studies ignored the special properties of chiral pesticides and the potential safety risks caused by the chiral enantiomers. It has been estimated that 25% of pesticides currently sold are chiral, and the proportion is estimated to be over 40% in China with increasing complex compounds registered for use [33]. In this study, due to the different toxicities of cyflumetofen enantiomers, we aimed to study the enantioselective degradation of cyflumetofen in apples under field conditions and evaluate the distribution pattern of this pesticide, which sheds light on its safe usage and accurate risk evaluation. The result from this study may provide more accurate information for evaluating the apple safety induced by cyflumetofen.

## 2. Results and Discussion

### 2.1. Method Validation

Apple samples planted in control plots were used as field blanks. Working solution mixtures of cyflumetofen were added to blank samples at three concentration levels (50 μg/kg, 500 μg/kg, and 1000 μg/kg), which were used for the method recovery assay and detection limit evaluation. The blanks were treated in the same way as other samples throughout the whole analysis.

#### 2.1.1. UPC^2^ System Performance

To obtain good resolution within a reasonable analysis time, Trefoil AMY1 (150 mm × 2.1 mm i.d., 2.5 μm particle size; Waters, MA, USA) was used as a chiral chromatographic column, and separation was performed for five minutes using isocratic elution with solvents A (CO_2_) and B (isopropanol) at a ratio of 96.5:3.5 (*v*/*v*) and a flow rate of 1.2 mL/min. As shown in Figure 1, good resolution and peak shapes were obtained for the peaks corresponding to the two enantiomers. Subsequently, to improve the separation of the two enantiomers, isopropanol was selected to combine with liquid CO_2_. These co-solvents were selected as “greener” based on their significantly lower toxicity and limited threat to the natural environment [34,35]. 

#### 2.1.2. Linearity, Limit of Detection (LOD), and Limit of Quantitation (LOQ)

Method linearity was determined by linear regression analysis of both standard solution and matrix-matched calibration curves, with the utilized cyflumetofen concentrations equal to 10–5000 μg/L. Satisfactory linearity was observed in all cases (*R*^2^ > 0.9990). 

LOD was defined as the lowest detectable concentration with a signal-to-noise ratio of three, which is equal to 2.8–4.7 µg/L for both (−)- and (+)-cyflumetofen in all apple parts. According to the SANTE/11813/2017 [36] guideline of the European Commission, the LOQ is defined as the lowest spiked level used for method validation, which equalled 10 µg/L in this study.

#### 2.1.3. Matrix Effect, Precision, and Accuracy

**Figure 2:**
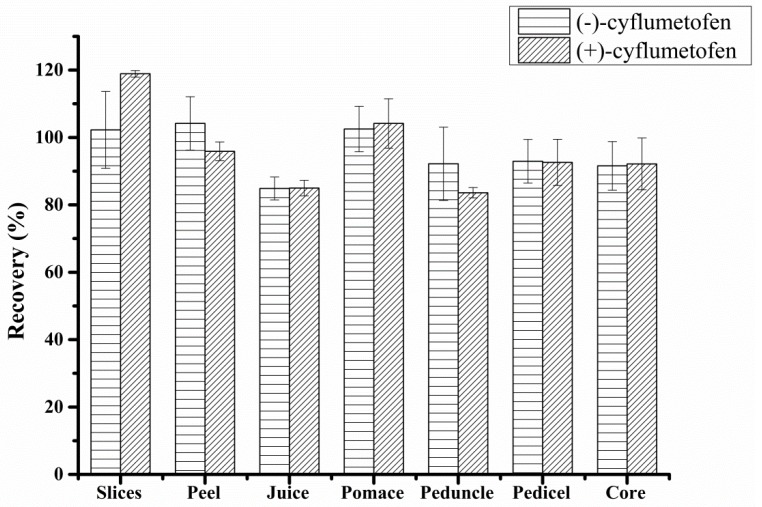
Cyflumetofen recoveries in seven studied matrices at three spiking levels.

The developed method was validated by determining cyflumetofen recoveries and relative standard deviations (RSDs). Since matrix components can affect the ionisation of target compounds, the matrix effect on MS/MS (MRM mode) detection was determined by comparing the standards in working and matrix-matched solutions. As shown in Figure 2, the recoveries observed at three concentration levels (50 μg/kg, 500 μg/kg, and 1000 μg/kg) equalled 83.6–118.9% based on acceptable RSDs of 1.1%–14.0%, which falls within the expected range. The values reported in Figure 2 are “means ± standard error of mean”.

#### 2.1.4. Uncertainty

Uncertainty is a basic characteristic of any measurement, and helps to assure the reliability of an analyst’s work [37]. In this study, the measurement uncertainty was determined for all compounds at three spiked levels using the bottom-up approach based on the in-house validation data in accordance with EURACHEM/CITAC [38]. The main sources of uncertainty were identified and quantified, and the combined uncertainty (*U*_c_) was calculated using the equation below:

*U*_c_ = (*U*_1_^2^ + *U*_2_^2^ + *U*_3_^2^ + *U*_4_^2^)^1/2^

where, *U*_1_ is the relative standard uncertainty of standards and stock solutions, *U*_2_ is the relative standard uncertainty of the calibration step, *U*_3_ is the relative standard uncertainty of repeatability, and *U*_4_ is the relative standard uncertainty of accuracy. The expanded uncertainty (*U*_exp_) was obtained from the combined uncertainty by multiplying by a coverage factor *k* = 2 to ensure a level of confidence of 95%, which is shown in the equation below

*U*_exp_ = k × *U*_c_

The results obtained for each individual source of uncertainty, the combined uncertainty *U*_c_, and the expanded uncertainty *U*_exp_ are summarized in Table 1. The *U*_exp_ values were 6.0% and 8.5% for (−)-cyflumetofen and (+)-cyflumetofen, respectively, which yielded an average value of 7.3%. This uncertainty is distinctly less than the maximum threshold value of 50% recommended by SANTE/11813/2017 [36], which clearly demonstrates the fitness for the developed method.

### 2.2. Stereoselective Degradation of Cyflumetofen

**Figure 3:**
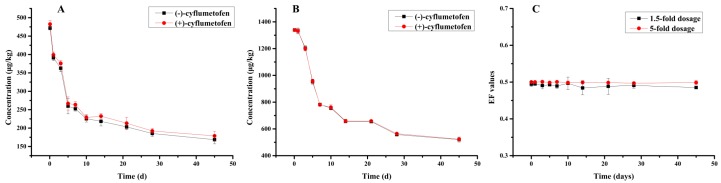
Degradation of cyflumetofen under field conditions, (**A**) 1.5-fold applied dosage; (**B**) 5-fold applied dosage; (**C**) EF value.

Apple trees were sprayed with 1.5-fold and 5-fold of highest dosage recommended of cyflumetofen SC by the manufacturer under field conditions. The degradation of cyflumetofen enantiomers in apples were demonstrated to follow the pseudo-first-order kinetic model. As shown in Figure 3A,B, the concentrations of cyflumetofen stereoisomers were maximal after 2 h and the concentration of (−)-cyflumetofen and (+)-cyflumetofen were achieved as 471.88 μg/kg and 482.50 μg/kg with 1.5-fold applied dosage and 1339.48 μg/kg and 1338.53 μg/kg with 5-fold applied dosage. It was then decreased to almost 50% of the initial values after 10 days. Under the 1.5-fold applied dosage condition, the concentrations of (+)-cyflumetofen enantiomer increased again to 232.40 μg/kg at 14 days. This phenomenon was also observed by Sun et al. [9] in which the two enantiomers of cyflumetofen were increased at 7 days. After 14 days, (−)-cyflumetofen and (+)-cyflumetofen under 1.5-fold applied dosage then gradually declined over time to 168.56 μg/kg and 178.82 μg/kg at 45 days with degradation rates of 63.8% and 62.5%, respectively (see Figure 3A). Under 5-fold applied dosages, the two enantiomers gradually declined with time to 520.44 μg/kg and 522.99 μg/kg at 45 days with degradation rates of 61.2% and 60.1%, respectively (see Figure 3B).

*T*_1/2_, a crucial indicator of pesticide efficacy and pollution [29,39], was calculated using Equation (2) (see Table 2), with the values for 5-fold applied dosage (+)-cyflumetofen and (−)-cyflumetofen equal to 22.23 days and 22.13 days, respectively. For 1.5-fold applied dosage, the respective values were determined as 23.64 days and 22.42 days, which indicates that dilution did not exhibit a significant effect on the cyflumetofen half-life. In addition, half-lives of cyflumetofen in apples were longer than in citrus and strawberries which was reported to be 19.77 days and 9.36 days, respectively [9,27].

EF is a straightforward measure of enantioselectivity since it is defined by Equation (3) [40]. Notably, cyflumetofen EF values during the field trial were maintained at ~0.50 (see Figure 3C), which indicates that both enantiomers exhibit very similar degradation behaviors. Eventual experiments showed that the degradation of (−)-cyflumetofen was slightly faster than that of (+)-cyflumetofen, with the corresponding enantioselectivity being very weakly pronounced, which was ascribed to the absence of a chiral degradation environment.

Microbial action is known to play an important role in the degradation of malathion, which is a widely used chiral pesticide [41]. In this regard, the investigation of the field-to-table fate of cyflumetofen residues in apples appeared meaningful, which led us to study the fate of cyflumetofen enantiomers during apple processing, especially in the course of fermentation.

### 2.3. Distribution of Cyflumetofen in Apples

**Figure 4:**
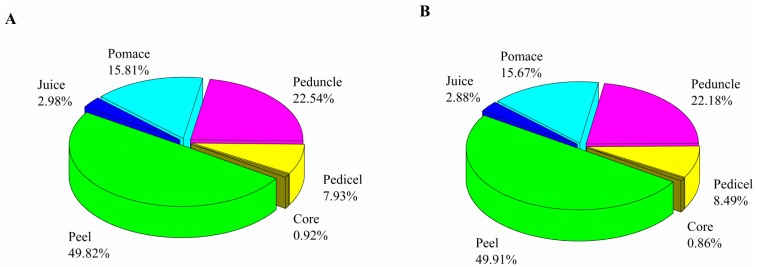
Distributions of cyflumetofen in apple samples, (**A**) (−)-cyflumetofen; (**B**) (+)-cyflumetofen.

Figure 4A,B reveals that both cyflumetofen enantiomers featured very similar distributions in apples since they are predominantly concentrated in the apple peel (50% of the total cyflumetofen content). The implies and implying that apples should be peeled prior to consumption. The obtained results were supported by Liu [28], who estimated the PF of the peeling process for cyflumetofen as 0.28. Han et al. showed that peeling and coring lead to the loss of 62.9% of spirotetramat-enol and 76.4% of spirotetramat from apples, with the PFs of coring and peeling estimated as 0.22 and 0.14, respectively [42]. Moreover, 22% of cyflumetofen was contained in the apple peduncle (a sag area easily accumulating pesticides), which highlights the importance of removing this part before apple consumption. In addition, the apple pomace also accounted for a considerable cyflumetofen fraction due to the poor water solubility of this pesticide. Therefore, safety issues need to be taken into account when apple pomace and apple peel are used for the extraction of polyphenols and flavonoids.

### 2.4. Dietary Intake Risk Assessment

The acceptable daily intake (ADI) of cyflumetofen per kg of body weight was determined to be 0.17 mg/kg bw/day on the European Food Safety Authority [43]. The STMRi in 1.5-fold dosage and 5-fold dosage apples is 0.22 mg/kg and 1.04 mg/kg, respectively. Characterizations of the population and apple consumption data were obtained from the 2002 Chinese National Nutrition and Health Survey (CNNHS) conducted via the 24-h recall method using a multistage and random cluster process. The average apple consumption in different subgroups was calculated by taking into consideration detailed information on total production, export, processing, and storage loss of apples in China [44]. The equivalent calculation was performed according to the proportion relationship of the consumption of fruits for different subgroups in the 2002 survey. Table 3 lists average body weights and apple consumption data in different age groups.

Table 4 lists the dietary intake risk assessment results of cyflumetofen for different age groups in urban and rural areas. Generally, kids were considered the most vulnerable group because they were particularly susceptible to the hazards associated with pesticides and tended to eat more apples on a body-weight basis [45,46,47]. From Table 4, we found that most of RQ % was more than 1 under 5-fold applied dosage, and this was because higher levels of applied pesticide dosage have more pesticide residue in the food. In addition, under 1.5-fold condition, we found higher health risks for kids compared with other age groups because the RQ % were 0.8 with rural male and more than 0.9 in urban male, rural female and urban female. This could be due to the consumption of fruits (including apple) in rural male of children, which were less than other children. Furthermore, exposure risks faced by teenagers, young adults, and seniors appeared to decline successively. In sum, the consumption of apples with residue of cyflumetofen did not pose a health risk to the population if the farmer applied the cyflumetofen under good agricultural practices.

## 3. Materials and Methods

### 3.1. Chemicals and Reagents

Analytical-grade cyflumetofen (97%) was purchased from Otsuka AgriTechno Co., Ltd. (Osaka, Japan), and commercial 20% cyflumetofen suspension concentrate (SC) was obtained from Jiangsu FMC Corporation (Jiangsu, China). Ultra-pure water was obtained using a Milli-Q system (Millipore, Burlington, MA, USA). HPLC-grade isopropanol, methanol, and acetonitrile were purchased from Fisher Scientific (Shanghai, China), which was in the form of formic acid (FA). Analytical-grade anhydrous MgSO_4_ and NaCl were purchased from Sinopharm Chemical Reagent Co., Ltd. Graphitised carbon black, primary secondary amine (PSA), and C_18_ were purchased from DIKMA (Beijing, China). Samples were filtered through a 0.22-µm organic filter membrane (Shanghai, China) before injection. 

Standard cyflumetofen stock solutions (1000 mg/L) were prepared in pure acetonitrile, and standard working solutions (0.01 mg/L, 0.05 mg/L, 0.1 mg/L, 0.5 mg/L, 1.0 mg/L, 3.0 mg/L, and 5.0 mg/L) for calibration curve construction were prepared in pure acetonitrile by serial dilution of the stock solution. Matrix-matched standard solutions were prepared at concentrations of 0.01 mg/L, 0.05 mg/L, 0.1 mg/L, 0.5 mg/L, 1.0 mg/L, 3.0 mg/L, and 5.0 mg/L by adding blank apple, apple juice, and apple cider extracts to each serially diluted standard solution. All sample and standard solutions were protected from light with aluminium foil and stored in a refrigerator at 4 °C until analysis. The working standard solutions underwent no degradation during a three-month storage.

### 3.2. Field Trials

Field experiments were performed at an experimental base (Beijing) from July to October 2016, with the experimental field surveyed and determined to be cyflumetofen-free before these experiments. Commercial 20% cyflumetofen SC was applied at the 1.5-fold and 5-fold of highest dosage recommended by the manufacturer of 0.20 and 0.67 active ingredient mg/kg to ensure sufficient pesticide primary deposit for the following degradation and processing studies. Target apples were sprayed to achieve an even distribution on the front and back sides of apple leaves, the fruit surface, trunk, and branches. Spraying was performed until the cyflumetofen suspension concentrate started to form droplets and drop down from the fruit surface. Two sprayings were performed using the above method. The interval between two applications was 10 days. Three kilograms of apple samples were picked at 0 (2 h), 1 day, 3 days, 5 days, 7 days, 10 days, 14 days, 21 days, and 28 days after the last spraying, and all the remaining apple samples were picked at 45 days after the last spraying. Then these apple samples were put into polyethylene bags, and transported to the laboratory, where some of these samples were used for studying cyflumetofen distributions. The other ones were chopped, mixed, divided into subsamples, and stored at −20 °C until analysis.

### 3.3. Distribution of Cyflumetofen in Apples

Apples were hand-peeled using a peeler, with the thickness of the removed skin equal to 0.2 cm [10]. The apple core, pedicel and peduncle were cut into pieces, and the remaining fruit was cored, peeled, cut into quarters, and put into an automatic juice extractor to produce juice and pomace.

### 3.4. Extraction and Purification

**Figure 5:**
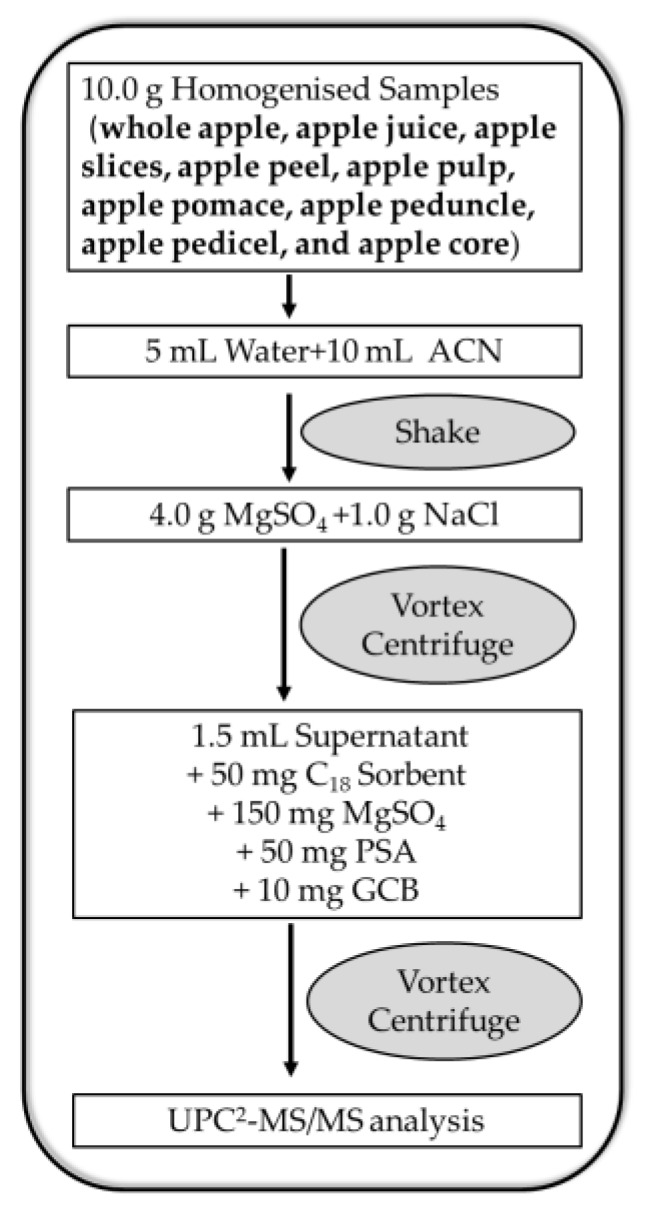
Diagram of analytical procedure including extraction and purification steps.

Figure 5 showed the extraction and purification steps of the analytical procedure. 10.0 g of homogenised samples were weighed into a 50-mL polypropylene centrifuge tube, and then charged with water (5 mL) and acetonitrile (10 mL) and mechanically shaken (SPEX Sample Prep, Metuchen, NJ, USA) for 5 min at 1200 strokes per minute. Next, anhydrous MgSO_4_ (4.0 g) and NaCl (1.0 g) were added to the mixture, which was followed by 3-min full-speed vortexing (XW-80A Vortex, Kirin Medical Instrument, Jiaxing, China) and 5 min centrifugation at 5000 rpm/min (TG16-WS, Xiangyi Centrifuge Machines, Changsha, China). Subsequently, 1.5 mL of the obtained supernatant was transferred to a 2-mL polypropylene centrifuge tube containing cleaning sorbent. In addition, the tube was vortexed at full speed for 2 min and centrifuged for 5 min, with 1 mL of the upper layer subsequently filtered through a 0.22 μm organic nylon membrane for UPC^2^-MS/MS analysis [23,29].

### 3.5. Chromatographic and Mass Spectrometric Conditions

An Acquity UPC^2^ system (Waters, Milford, MA, USA) equipped with a column manager, binary solvent manager, sample manager-FL, convergence manager, and a Waters 515 compensation pump was used for detection. Trefoil AMY1 (150 mm × 2.1 mm i.d., 2.5 μm particle size; Waters, Milford, MA, USA) was used as a chiral chromatographic column. Separation was performed for 5 min using isocratic elution with solvents A (CO_2_) and B (isopropanol) at a ratio of 96.5:3.5 (*v*/*v*) and a flow rate of 1.2 mL/min. The automated backpressure regulator pressure (ABPR) was set at 2000 psi, and 0.1% FA/MeOH (*v*/*v*) was employed as a post-column additive of the 515 compensation pump at a flow rate of 0.40 mL/min. The sample manager and column were maintained at 25 °C and 40 °C, respectively, and the sample injection volume was set at 1 μL [14].

Detection of cyflumetofen stereoisomers was performed by using a triple quadrupole Xevo-TQD mass spectrometer (Waters, Milford, MA, USA) operated in positive-ion ESI mode. The following acquisition parameters were utilized: source temperature = 150 °C, desolvation temperature = 500 °C, and capillary voltage = 3.5 kV. The gas consisted of collision gas (99.99% Ar) and nebuliser gas (99.95% N_2_) at a pressure of 2 × 10^−3^ mbar in a T-wave cell. Cone and desolvation gas flows equalled 50 L/h and 900 L/h, respectively. Species with *m*/*z* 448 and 173 were selected as cyflumetofen precursor and quantitative product ions, respectively. Multiple reaction monitoring (MRM) was selected as the scan mode [14].

### 3.6. Dissipation Kinetics and Enantioselectivity of Cyflumetofen

All experiments were repeated three times, and values were reported as the mean ± standard deviation. Analysis of variance was utilized to compare data between groups, with *p* values below 0.05 (*t*-test) considered statistically significant. All statistical analyses were performed in SAS (version 9.2, SAS Institute, Beijing, China). The half-life *T*_1/2_ (d) in apples was calculated according to the equations below.

*C* = *C*_0_*e*^−*kt*^ (1)

*T*_1/2_ = ln2/*k* (2)

where, *C* is the residue concentration (mg/kg) of cyflumetofen at time point t (day), *C*_0_ is the initial concentration (mg/kg) of the pesticide, and *k* is the cyflumetofen degradation rate constant (day^−1^).

Enantiomer fractions (EFs, ranging from 0 to 1 and equal to 0.5 for a racemic mixture) were used to evaluate the enantioselectivity of cyflumetofen degradation in apple samples. This was determined using the equation below.

(3) $$\mathrm{EF}=\frac{\mathrm{Concentration}\;\mathrm{of}\;\left(-\right)-\mathrm{cyflumetofen}}{\mathrm{Sum}\;\mathrm{of}\;\left(-\right)-\;\mathrm{and}\;\left(+\right)-\mathrm{cyflumetofen}\;\mathrm{concentrations}}$$

### 3.7. Dietary Intake Risk Assessment

The long-term dietary risk assessment of cyflumetofen in apple was calculated by using Equations (4) and (5).

NEDI = ∑ (STMRi × Ei × Pi ×Fi)(4)

RQ % = NEDI/(ADI × bw) × 100(5)

where NEDI is Estimated Daily Intake. STMRi is the supervised trials median residue level and the mean value of the final residue was used in this study. Fi is the apple per day consumption (kg/d). RQ is Risk Quotient. Ei and Pi refer to the factor for the edible part and the processing factor of a particular food product, respectively. In this study, the difference was not considered and both Ei and Pi were set to 1. ADI is the acceptable daily intake in mg/kg bw. bw refers to body weight in kg and in general; RQ % > 1 indicates the presence of unacceptable risk while RQ % < 1 indicates an acceptable risk. The risk is positively correlated to the RQ value [48].

## 4. Conclusions

In this paper, we investigated the distribution and enantioselective degradation of cyflumetofen in apples and, observed almost no differences in the behaviors of the two enantiomers. The degradation rate of (−)-cyflumetofen was only insignificantly faster than that of (+)-cyflumetofen. Importantly, cyflumetofen was concentrated in the apple peel and pomace, which implies that safety considerations need to be taken into account when these parts are used for bioactive molecule extraction. Overall, the current study shed new light on the stereoselective dissipation and distribution of cyflumetofen, which contributes to a better understanding of this novel acaricide and provides more accurate information for comprehensive risk assessment and regulatory decisions related to the use of cyflumetofen in agricultural production. After the long-term dietary risk assessment, the consumption of apples with residue of cyflumetofen did not pose a health risk to the population if the farmer applied the cyflumetofen under satisfactory agricultural practices.

The result of this study showed that there is urgent need to monitor the pesticide residues to standardize the application doses especially for chiral pesticides. Pest management is one of the major inputs in agricultural production the chiral pesticide needs great attention to economize the production, to provide safe foods and to lower the medical expenses for treatment of resulting ailments. As for agricultural use, potential substitution of rac-cyflumetofen with isomer-enriched compound should take into consideration to reduce the residues in plant and reduce the negative effect on environment and human health. In this study, (−)-cyflumetofen was insignificantly faster than that of (+)-cyflumetofen, and Sun et al., [9] found that (+)-cyflumetofen was much lower in cytotoxicity when compared with (−)-cyflumetofen. Therefore, (+)-cyflumetofen might be developed as the substitution of cyflumetofen racemate if it is more efficient to target organisms than (−)-cyflumetofen. In addition, the results would further enable research on health risk characterization from realistic dietary exposures to pesticide residues, which would enable the settlement of MRLs or processing factors for pesticide residues in food products, and further enable the optimization of food technology processes with regard to pesticide residues dissipation. Overall, the findings of this study provide some implications for better understanding of this new pesticide and provide some relevance to the registration and usage advice. In the future, we plan to investigate the fate of cyflumetofen enantiomers during apple processing (focusing on the fermentation) to provide some implications for better environmental and ecological risk assessment.

## Figures and Tables

**Table 1:** Uncertainty (*U*_c_) and expanded uncertainty (*U*_exp_) in apple matrices for (−)-cyflumetofen and (+)-cyflumetofen at 50, 500, and 1000 μg/kg.

Uncertainty	(−)-cyflumetofen	(+)-cyflumetofen
*U*_1_	0.0025	0.0025
*U*_2_	0.0202	0.0305
*U*_3_	0.0045	0.0038
*U*_4_	0.0218	0.0293
*U*_c_	0.0301	0.0425
*U*_exp_ (%)	6.0	8.5

**Table 2:** Degradation kinetic constant (k), Half-life (*T*_1/2_) and correlation coefficient (*R*^2^) values of cyflumetofen in apple.

Sample	Enantiomer	K × 10^−2^ (day^−1^)	*T*_1/2_ (day)	*R* ^2^
Apple (1.5-fold dosage)	(−)-cyflumetofen	4.01	22.42^a^	0.7938
	(+)-cyflumetofen	3.32	23.64^a^	0.7662
Apple (5-fold dosage)	(−)-cyflumetofen	3.17	22.13^a^	0.7468
	(+)-cyflumetofen	2.70	22.23^a^	0.7736

**Table 3:** Characterization of the population and dietary intake of apples.

Subpopulation	Age	Average Body Weight (kg)				Consumption of Apples (g·day^−1^)			
		Female		Male		Female		Male	
		Urban	Rural	Urban	Rural	Urban	Rural	Urban	Rural
Kids	2–6	17.4	15.5	18.3	16.1	126.3	109.5	134.1	99.4
Children	7–13	34.1	29.4	35.3	29.6	154.9	96.6	147.0	101.4
Teenagers	14–17	51.0	47.8	56.2	50.4	209.0	108.3	177.1	91.5
Adults	18–59	56.0	54.0	65.7	60.5	162.2	84.0	118.3	73.6
Seniors	≥60	54.9	48.8	63.7	55.9	132.8	42.3	142.6	45.9

**Table 4:** Dietary intake risk assessment of cyflumetofen in whole apple.

Subpopulation	Age	RQ %							
		1.5-Fold				5-Fold			
		Female		Male		Female		Male	
		Urban	Rural	Urban	Rural	Urban	Rural	Urban	Rural
Kids	2–6	0.94	0.91	0.95	0.80	4.44	4.32	4.48	3.78
Children	7–13	0.59	0.43	0.54	0.44	2.78	2.01	2.55	2.10
Teenagers	14–17	0.53	0.29	0.41	0.23	2.51	1.39	1.93	1.11
Adults	18–59	0.37	0.20	0.23	0.16	1.77	0.95	1.10	0.74
Seniors	≥60	0.31	0.11	0.29	0.11	1.48	0.53	1.37	0.50

## Abbreviations

The following abbreviations are used in this manuscript.

UPC^2^-MS/MS	Ultra-performance convergence chromatography coupled with triple quadrupole mass spectrometry
PF	Processing factor
SC	Suspension concentration
PSA	Primary secondary amine
MRM	Multiple reaction monitoring
EF	Enantiomeric fraction
LOD	Limit of detection
LOQ	Limit of quantitation
ABPR	Automated back pressure regulator
FA	Formic acid
RSD	Relative standard deviations
ADI	Acceptable daily intake
NEDI	National estimated daily intake
STRM	Standardized test residue median
RQ	Risk quotient
